# The Safety and Efficacy of Dietary Epigallocatechin Gallate Supplementation for the Management of Obesity and Non-Alcoholic Fatty Liver Disease: Recent Updates

**DOI:** 10.3390/biomedicines13010206

**Published:** 2025-01-15

**Authors:** Ruike Yan, Yanli Cao

**Affiliations:** Department of Endocrinology and Metabolism, Institute of Endocrinology, NHC Key Laboratory of Diagnosis and Treatment of Thyroid Diseases, The First Affiliated Hospital of China Medical University, Shenyang 110001, China; yanruike0130@163.com

**Keywords:** epigallocatechin gallate, obesity, non-alcoholic fatty liver disease, metabolic disease

## Abstract

Epigallocatechin gallate (EGCG) is the predominant bioactive catechin in green tea, and it has been ascribed a range of beneficial health effects. Current increases in obesity and non-alcoholic fatty liver disease (NAFLD) rates represent a persistent and burdensome threat to global public health. While many clinical studies have demonstrated that EGCG is associated with positive effects on various health parameters, including metabolic biomarkers, waist circumference, and body weight when consumed by individuals affected by obesity and NAFLD, there are also some reports suggesting that it may entail some degree of hepatotoxicity. The present review provides a comprehensive summary of the extant clinical findings pertaining to the safety and effectiveness of EGCG in managing obesity and NAFLD, with a particular focus on how treatment duration and dose level affect the bioactivity of this compound.

## 1. Introduction

Obesity is a serious and growing threat to global public health and human longevity [1]. Based on recent statistics, the prevalence of obesity among women globally has risen from 8.8% (8.5–9.1%) in 1990 to 18.5% (17.9–19.1%) in 2022, with a corresponding increase from 4.8% (4.6–5.0%) to 14.0% (13.4–14.6%) among men [2]. Treating obesity is a costly process, and healthcare costs tend to rise in parallel with increasing body mass index (BMI) values. By 2035, the global costs associated with managing excessive weight and obesity are forecast to reach USD 4.32 trillion [3,4]. In addition to shortening the life expectancy of affected individuals, obesity also increases the risk of cardiovascular disease and diabetes [5,6]. Rising obesity rates are also closely associated with rising non-alcoholic fatty liver disease (NAFLD) incidence rates, with a 3.5-fold greater likelihood of NAFLD among obese individuals relative to individuals of normal body weight (RR = 3.53, 95% CI 2.48 to 5.03, *p* < 0.001) [7]. The prevalence of NAFLD is rising to epidemic proportions, with patients exhibiting a range of phenotypes along a spectrum from simple steatosis to non-alcoholic steatohepatitis (NASH), which can lead to cirrhosis and hepatocellular carcinoma [8]. In 2022, NAFLD was estimated to affect 32–34% of the global population [9].

While obesity is associated with a wide range of health risks, achieving weight loss and maintaining any reductions in body weight remains extremely difficult [10]. Myriad approaches are used to treat obesity, including dietary modifications, lifestyle changes, behavioral therapy, nutritional supplementation, pharmacologic treatment, and, if appropriate, surgery [11]. The low adherence rates for dietary modification, lifestyle adjustment, and behavioral intervention-focused approaches to managing obesity tend to yield low rates of success. While pharmacologic and surgical interventions can be effective, they are also costly and expose patients to the potential for adverse events [12]. It has been proposed that nutritional supplementation may represent a safer yet effective approach to managing obesity, providing patients with the opportunity to lose weight and restore metabolic health with fewer side effects relative to surgery or pharmacologic treatment. Much like obesity, there are few effective options available for the treatment of NAFLD and NASH, making the management of this disease challenging [13]. Rezdiffra, the first globally authorized treatment for NASH, was approved by the US FDA in 2024 [14]. While efficacious, this drug is associated with the potential for nausea, diarrhea, and other adverse events, and its utilization is also contraindicated in certain patient populations. The therapeutic efficacy of Rezdiffra treatment is also limited, with only ~30% of patients presenting with improved NASH symptoms [15]. Under current clinical guidelines, phase 3 clinical trials have been authorized for biopsy-confirmed NASH patients [16], involving trials focused on the therapeutic effects of vitamin E [17], pioglitazone [17,18], Selonsertib [19], obeticholic acid [20], Cenicriviroc [21], and Elafibranor [22]. In line with the fact that NAFLD has been linked with metabolic syndrome, exercise, dietary changes, and other lifestyle modifications can reduce hepatic fat levels and limit hepatic fibrosis. Most patients still face difficulties sustaining weight loss, and a majority of these individuals are unable to maintain the associated lifestyle changes over extended periods [23,24,25]. There is therefore a pressing need to define reliable approaches to treating NAFLD and initiating these interventions at early time points to more effectively slow or prevent progression to NASH.

Green tea is rich in bioactive compounds and remains an extremely popular beverage globally, especially in Asian countries. It is rich in vitamins, carbohydrates, minerals, pigments, proteins, polyphenols, and caffeine, all of which have been proposed to contribute to the health-promoting properties of this beverage [26]. Green tea-derived polyphenols, particularly catechins, are believed to mediate its therapeutic anti-inflammatory, antioxidant, and metabolic effects, as demonstrated in a series of published clinical studies [27,28,29]. Epigallocatechin gallate (EGCG) is highly bioactive and represents the predominant catechin in green tea, making up 20–30% of green tea leaves by dry weight [30]. EGCG presents with a plethora of beneficial effects, such as antioxidant, anticancer, and anti-inflammatory properties [31,32,33]. As a common plant-derived compound, EGCG can be obtained cost-effectively and it is generally recognized as safe [34]. In recent years, an increasing body of research has demonstrated its ability to support weight reduction and retard NAFLD progression. Animal studies have demonstrated that EGCG can significantly reduce body weight in mice fed a high-fat diet and positively influence their lipid profile, including triglyceride, cholesterol, high-density lipoprotein cholesterol (HDL-C), and low-density lipoprotein cholesterol (LDL-C) levels [35]. In high-fat diet-induced obese rats, EGCG improved serum glucose levels, mitigated inflammation, and reduced oxidative stress associated with aging [36]. Furthermore, EGCG has been shown to enhance liver health by reducing hepatic fat accumulation, oxidative damage, and inflammatory responses in high-fat-fed rats [37]. Clinical studies also suggest that EGCG can aid in weight reduction and metabolic improvement in obese patients while slowing the progression of NAFLD [38,39,40]. Variability in dosage, intervention duration, and study outcomes has been noted in existing research. Clinical studies investigating EGCG intervention for obesity and NAFLD have utilized daily doses ranging from 150 mg to 900 mg. However, studies have suggested that EGCG may pose risks to liver health, with potential concerns related to dosage. Currently, there is a limited number of comprehensive reviews summarizing the clinical evidence for EGCG in the treatment of NAFLD. The determination of safe and effective dosages, as well as the therapeutic potential of EGCG for managing obesity and NAFLD, remain important areas for further investigation.

Considering the association between obesity and NAFLD, this review aims to summarize the current evidence on the utilization of green tea extracts, specifically EGCG, in the management of obesity or NAFLD. It seeks to evaluate the therapeutic efficacy and safety of EGCG across different dosages and intervention durations.

## 2. EGCG: A Brief Overview

EGCG has a core structure consisting of four rings that are the primary site of its bioactivity owing primarily to reactive phenolic hydroxyl groups present on its core rings [41]. The reported therapeutic effects of EGCG include antitumor, anti-mutagenic, anti-inflammatory, and antioxidant activities, and it has also been linked to reductions in body weight and visceral fat levels [33,42,43,44,45].

### 2.1. Sources of EGCG

EGCG is primarily isolated from green tea, wherein it comprises ~40% of the total catechins [46]. Smaller amounts of EGCG can also be isolated from black tea, white tea, and oolong tea, while trace amounts are present in certain beans, nuts, and fruits including berries, plums, and apples [33,47,48].

### 2.2. Potential Biological Effects of EGCG in the Treatment of Obesity and NAFLD

EGCG can reduce lipogenesis by activating Adenosine Monophosphate-Activated Protein Kinase (AMPK). For example, Santana et al. found that after 8 weeks, compared to high-fat diet + EGCG-fed mice, high-fat diet + placebo-fed mice showed increased body weight. Additionally, when compared to the control diet + placebo group, the control diet + EGCG group showed a 16% reduction in body weight and a 32% decrease in serum triglyceride levels. Compared to the control diet + placebo group, the control diet + EGCG group exhibited increased phosphorylation of AMPK and hormone-sensitive lipase in both epididymal and retroperitoneal adipose tissues. Furthermore, increased phosphorylation of acetyl-CoA carboxylase was observed in both types of adipose tissues [49]. In vitro studies have shown that EGCG activates AMPK and reduces lipid accumulation in *HepG2* cells. Activation of AMPK shifted some free fatty acids (FFAs) toward oxidation, away from lipid and triglyceride storage, and suppressed hepatic gluconeogenesis [50]. These findings suggest that EGCG may alleviate body weight and delay NAFLD progression by promoting fat breakdown and reducing lipid accumulation. Lee et al. reported that in the epididymal white adipose tissue of mice fed EGCG, there was a significant reduction in the mRNA expression of adipogenic genes, including *peroxisome proliferator-activated receptor-gamma* (*PPAR-γ*), *CCAAT enhancer-binding protein-alpha* (*C/EBP-α*), *sterol regulatory element-binding protein-1c* (*SREBP-1c*), *adipocyte fatty acid-binding protein* (*aP2*), *lipoprotein lipase* (*LPL*), and *fatty acid synthase* (*FAS*). In contrast, the mRNA levels of *carnitine palmitoyltransferase-1* (*CPT-1*), *uncoupling protein 2* (*UCP2*), and *lipolytic genes like hormone-sensitive lipase* (*HSL*) and *adipose triglyceride lipase* (*ATGL*) were significantly elevated [51]. This suggests that EGCG’s effects on weight reduction are mediated by multiple genes. In addition to reducing body weight by promoting fat breakdown and decreasing fat synthesis, EGCG can also affect the gastrointestinal absorption of lipids and proteins. For example, in a study with mice, EGCG dose-dependently reduced food digestibility and increased fecal excretion. With a 13C-triglyceride-enriched diet, EGCG supplementation increased the 13C levels in the feces. Moreover, the study also found that EGCG could downregulate genes related to hepatic fat synthesis, including *Acetyl-CoA carboxylase* (ACC), *FAS*, and *Stearoyl-CoA desaturase 1* (*SCD1*) [52]. Studies have shown that EGCG can increase the lipid content in the feces of mice, which is related to its effect on pancreatic lipase [53]. EGCG can reduce liver weight and triglyceride levels in high-fat-fed mice, decreasing lipid accumulation in hepatocytes [54]. Additionally, Santamarina et al. found that EGCG seems to enhance mitochondrial complex chain activity, thereby increasing lipid oxidation and preventing liver fat degeneration [55]. EGCG can also improve the expression of inflammatory mediators in liver adipose tissue. Yuan et al. found that EGCG significantly reduced the levels of NF-κB protein in the liver of rats fed a high-fat diet [36], which may help prevent the progression of simple fatty liver to NASH. EGCG can also modulate the gut microbiota. EGCG significantly increased the abundance of *Dubosiella* and *Akkermansia* in mice fed a high-fat diet, which is crucial for alleviating obesity [56]. Overall, EGCG treatment for obesity and NAFLD primarily exerts its effects by reducing lipogenesis, promoting lipolysis, inhibiting lipid absorption, and alleviating inflammatory responses.

### 2.3. EGCG Absorption and Bioavailability

EGCG absorption primarily occurs in the jejunum and ileum wherein it diffuses across the epithelium without any concomitant hydrolysis or decoupling [57]. Despite its advantageous health effects, EGCG and other catechins are associated with drawbacks that include poor stability [58]. Indeed, when incubated for 1 h in simulated intestinal fluid (pH 7.4), roughly 80% of EGCG is degraded. Studies of ileal fluid samples collected 24 h after administering green tea extract (200 mg) yielded catechin recovery rates between 39% and 48% of the ingested amount [59]. The recovery rate for EGCG is comparatively high at ~60% [60].

In both humans and rats, EGCG oral bioavailability has been found to be fairly low at just 0.1–0.3% [61]. This poor availability is attributed to the presence of polyphenolic hydroxyl groups within EGCG that are subject to reactions that include methylation, sulfonation, glucuronidation, and cysteine conjugation [62].

## 3. Clinical Overview of the Effects of EGCG on Obesity and NAFLD

### 3.1. The Impact of Supplemental EGCG Intake on Overweight and Obese Individuals

#### 3.1.1. Body Weight and Glycolipid Metabolism Responses to Low- or Medium-Dose EGCG

Table 1 shows an overview of clinical studies published to date in which researchers have examined the impacts of EGCG on overweight and obese individuals. An investigation by Kovacs et al. found that daily intake of green tea extract (323 mg/day of EGCG, 104 mg/day of caffeine) for three months had no significant effect on body weight [63]. A further study similarly determined that daily EGCG intake (300 mg/day) for three months failed to positively impact the body weight, fat metabolism, fat content, or energy balance of premenopausal obese women [38]. Despite the negative findings in both studies, the majority of the studies reviewed here still suggest that supplementation with low doses of EGCG over a three-month period can yield beneficial effects.

For example, Zhang et al. studied the impact of EGCG on blood lipid levels and hepatic function among males who were overweight or obese, administering green tea extract (150 mg/day of EGCG) or placebo control to 24 subjects for 12 weeks. They determined that participants receiving green tea extract exhibited markedly reduced LDL-C and total cholesterol (TC) levels relative to placebo controls at the end of this period [64]. Suliburska et al. further found that supplemental EGCG capsule intake (208 mg/day) for three months significantly decreased the body weights, waist circumferences, and LDL-C, TC, and triglyceride (TG) levels of obese participants [65]. Bogdanski et al. found that the daily intake of green tea extract capsules (208 mg/day of EGCG) for three months was associated with markedly reduced TC, LDL-C, and TG levels together with increased HDL-C levels relative to placebo in hypertensive obese patients. These changes also coincided with decreases in blood sugar, insulin, and fasting glucose levels, as well as improved insulin resistance, significantly decreased TNF-α and CRP levels, and the overall augmentation of patient antioxidant status [66]. The above study demonstrated that supplementation with EGCG for approximately three months can significantly improve lipid levels in overweight and obese individuals. This effect is attributed to EGCG’s ability to enhance lipid metabolism. Animal studies have shown that EGCG increases the expression of proteins such as fatty acid binding protein-1 (FABP1), long-chain acyl-CoA synthetase 1 (ACSL1), and carnitine palmitoyltransferase II (CPT2), which promote fatty acid transport and oxidation. Additionally, EGCG reduces the expression of acetyl-CoA carboxylase (ACC1) and fatty acid synthase (FAS), both of which are involved in fatty acid synthesis [36].

It was also found that the intake of a mixture of green tea and caffeine (EGCG, 270 mg/day; caffeine, 150 mg/day) for three months by participants who were overweight or moderately obese led to reduced body weights, waist circumferences, body fat, and respiratory quotients (RQs), while also increasing energy expenditure at rest [67]. In their randomized controlled trial enrolling overweight postmenopausal women with class I obesity, Rondanelli et al. found that the administration of a tea extract (300 mg/day, containing ≥13% EGCG) for 60 days resulted in significant reductions in CRP and insulin levels, enhanced fat oxidation, improved waist circumference, and enhanced resting energy expenditure relative to placebo control [68]. These two studies demonstrated that EGCG supplementation increased resting energy expenditure in overweight and obese participants, with a significant impact on anthropometric measurements. It has been reported that EGCG promotes fat oxidation (FOX) and weight loss by enhancing resting energy expenditure and reducing RQ. Additionally, this meta-analysis also indicated that EGCG supplementation led to a reduction in waist circumference [69]. Together, the above results suggest that the supplemental intake of EGCG at doses of 150–300 mg/day by obese individuals for approximately 3 months may help improve lipid levels and lower fasting blood glucose, and might improve anthropometric measurements.

Basu et al. found that obese individuals who drank four cups of green tea (440 mg/day of EGCG) or took two green tea extract capsules (460 mg/day of EGCG) per day for 8 weeks exhibited significant reductions in body weight and BMI. In these same individuals, EGCG intake led to pronounced reductions in circulating levels of the oxidative stress markers malondialdehyde (MDA) and hydroxynonenal (HNE), consistent with the mitigation of systemic lipid peroxidation [70]. Alves Ferreira et al. determined that the daily supplemental intake of 1 g of green tea extract (560 mg/day of total polyphenols) for 12 weeks by overweight women without diabetes markedly improved fasting blood glucose and lipid profiles, outperforming daily metformin treatment (1 g/day) [71]. Quinhoneiro et al. determined that when women with class III obesity consumed supplemental EGCG (450 mg/day) for 8 weeks, the *UCP3* gene associated with enhanced energy expenditure and reduced fat accumulation was upregulated. Even with these changes at the molecular level, however, the body weight of study participants was not significantly impacted by EGCG treatment [72]. Studies have shown that EGCG can reduce body weight in mice fed a high-fat diet by upregulating *UCP3* mRNA levels [73]. The study only observed an upregulation of *UCP3* levels without resulting in weight loss, possibly due to insufficient observation duration. Relatively few studies have thus explored the influence of supplemental EGCG doses in the 400–600 mg/day range on obesity, and differences in follow-up durations and analyzed outcomes are evident across these studies. At a high level, the evidence that is available suggests that EGCG exhibits antioxidant activity, can reduce levels of blood sugar, and may enhance energy expenditure.

#### 3.1.2. The Responses of Body Weight and Glycolipid Metabolism to High-Dose EGCG Treatment

Expanding on the above results, there have also been several studies specifically examining the impact of high-dose EGCG intake on obese individuals. In their study of 115 women with central obesity, Chen et al., for example, administered 856.8 mg/day of EGCG to a subset of these participants and observed markedly lower body weights, BMI values, and waist circumferences among EGCG-treated individuals relative to those in the placebo control group [74]. In contrast, Brown et al. found that the daily supplemental intake of 800 mg/day of EGCG for eight weeks failed to impact the glucose tolerance, insulin secretion, or insulin sensitivity of overweight/obese men relative to placebo controls, although it did reduce levels of diastolic blood pressure [75]. Dostal et al. reported significantly lower insulin levels among obese postmenopausal women with baseline fasting insulin levels ≥ 10 µIU/mL following high-dose EGCG treatment (843 mg/day) relative to placebo control. Study participants with homozygosity for a high-activity (G/G) variant of the *catechol-O-methyltransferase* (*COMT*) gene also presented with markedly lower adiponectin levels relative to individuals with the low-activity (A/A) genotype [76]. In a separate report, however, Dostal failed to observe any improvements in plasma leptin, ghrelin, or adiponectin levels among overweight postmenopausal women following daily EGCG treatment (834 mg/day) for 11–12 months [77]. Dostal et al. similarly found that the daily supplemental consumption of EGCG from green tea (843 mg/day) for 12 months had no impact on satiety, leptin, adiponectin, or ghrelin levels among postmenopausal women [78]. Huang et al. conducted a study of 36 overweight or obese women with elevated levels of LDL-C who were given 856.8 mg/day of ECGC for six weeks. Relative to placebo treatment, EGCG consumption led to markedly decreased LDL-C levels together with significantly elevated leptin levels [79]. Nicoletti et al. demonstrated that the daily supplemental intake of EGCG (901.4 mg/day) by severely obese women for 8 weeks resulted in the upregulation of *rapamycin-insensitive companion of mTOR* (*RICTOR*) and *hypoxia inducible factor 1 alpha* (*HIF1-α*), whereas *phosphoinositide-3-kinase* (*PI3K*) expression was unaffected. In the same study, EGCG also failed to reduce the weight of enrolled participants [80]. A randomized controlled trial indicated that, compared to the low dose (200 mg/day), a high dose (600 mg/day) of EGCG did not increase fat oxidation [81]. The effect of EGCG on body weight or metabolism may not necessarily be better with higher doses. The majority of these reports also suggest that EGCG supplementation is insufficient to reduce body weight, BMI, or other body measurements among obese subjects, even when administered at high doses. Moreover, high doses of EGCG may pose a risk of liver damage, which will be discussed in Section 4.

#### 3.1.3. The Synergistic Effects of EGCG and Other Polyphenols

Several studies to date have investigated possible combinatory or synergistic interactions between EGCG and other polyphenols in overweight or obese individuals. For instance, Most et al. noted no significant changes in overall body fat, energy expenditure, or plasma metabolite profiles following the supplemental intake of 282 mg of EGCG and 80 mg of resveratrol for three months [82]. This same combined polyphenol regimen, however, lowered the levels of factors involved in pathways linked to inflammation, immunity, and energy metabolism when suppressing apoptotic activity [83]. Supplemental EGCG and resveratrol intake among overweight or obese males has also been shown to be associated with significant decreases in *Bacteroidetes* and *Faecalibacterium prausnitzii* abundance within the gut microbiome. Baseline *Bacteroidetes* abundance was also demonstrated to predict increases in fat oxidation among these male participants in response to combined polyphenol supplementation, whereas these responses were absent among female participants [84]. Together, these results suggest that a 3-month supplemental EGCG and resveratrol treatment course can alter metabolic parameters and the composition of the gut microbiome in obese males, although these effects may be gender-specific. This information is crucial as it lays the foundation for further exploration of whether combining EGCG with other polyphenols could yield better results than using EGCG alone as a monotherapy.

#### 3.1.4. The Synergistic Effects of EGCG and Exercise

In their study exploring the potential additive benefits of green tea extract on exercise-induced bodily changes, Bagheri et al. separated 30 overweight women into three groups, including a placebo-treated group with no exercise training, a placebo-treated group that underwent endurance training, and a green tea extract-treated group that underwent endurance training. Participants in the lattermost group took 500 mg of green tea extract (15 mg caffeine, 45% EGCG) daily. Following an 8-week intervention period, markedly higher levels of adiponectin together with decreased high-sensitivity CRP (hs-CRP) in endurance training + green tea extract participants relative to the two other study groups, whereas IL-6 and irisin levels were comparable across groups [85]. Intriguingly, the administration of this same interventional approach to obese middle-aged men revealed that the combination of endurance training and green tea extract intake significantly reduced IL-6 levels and significantly reduced irisin levels, whereas TNF-α levels were unaffected by such treatment in either study [86]. These results suggest that there may be gender-specific effects on the EGCG treatment of obese patient populations. Hill et al. also demonstrated that daily EGCG supplementation (300 mg) together with regular exercise for 12 weeks led to marked reductions in blood glucose and resting heart rate levels compared to placebo combined with exercise, without any concomitant changes in body weights, waist circumferences, waist-to-hip ratios, total body fat, or abdominal fat [87]. More rigorous studies are needed to investigate the potential synergistic effects of combining EGCG with exercise.

**Table 1:** Clinical studies of the effects of epigallocatechin gallate (EGCG) on overweight and obese individuals.

Author	Country	Patients	Age	Dose, Intervention Period	Control	Outcomes
Mielgo-Ayuso et al. [38].	Spain	Obese pre-menopausal women.	19–49 years	Received a tablet of EGCG at 300 mg daily for 3 months (*n* = 43).	Placebo (*n* = 40)	Did not affect body weight, fat mass, energy and fat metabolism, homeostasis assessment model for insulin resistance, total cholesterol, LDL-cholesterol, triglycerides, and liver function markers
Kovacs et al. [63]	The Netherlands	Overweight and moderately obese subjects.	18–60 years	Received green tea extracts tablets containing 323 mg EGCG and 104 mg caffeine dailyfor 3 months (*n* = 51).	Placebo (*n* = 53)	Did not significantly affect weight gain in subjects on a very-low-energy diet intervention.
Zhang et al. [64].	China	Overweight and obese men.	42.5 years	Received 300 mg green tea extract containing 95% EGCG per day and four 60-min-sessions/week for 12 weeks (*n* = 12).	Placebo and four 60-min-sessions/week (*n* = 12).	Reduced the level of LDL-C, aspartate aminotransferase, HDL-C, and TC.
Suliburska et al. [65].	Poland	Obese subjects	30–60 years	Received a capsule containing 379 mg green tea extract containing 208 mg EGCG for 3 months (*n* = 23)	Placebo (*n* = 23)	Reduced body mass index, waist circumference, and levels of total cholesterol, LDL-cholesterol, and triglyceride, while enhancing total antioxidant level and zinc concentration in serum.
Bogdanski et al. [66].	Poland	Obese and hypertension subjects	30–60 years	Received a capsule containing 208 mg EGCG for 3 months (*n* = 28)	Placebo (*n* = 28)	Reduced blood pressures, fasting serum glucose, insulin levels, insulin resistance, Serum tumor necrosis factor α, C-reactive protein, the total and low-density lipoprotein cholesterol, and triglycerides; increased total antioxidant status and high-density lipoprotein cholesterol.
Westerterp-Plantenga et al. [67].	The Netherlands	Overweight and moderately obese subjects.	18–60 years	Received a green tea-caffeine mixture (270 mg EGCG + 150 mg caffeine per day) for 3 months (*n* = 38).	Placebo (*n* = 38)	Reduced body weight, waist, respiratory quotient, and body fat, while increasing resting energy expenditure.
Rondanelli et al. [68].	Italy	Overweight post-menopausal and class I obese women.	60.57 years	Received 300 mg Greenselect^®^ Phytosome^®^ containing more than 13% EGCG per day for 60 days. (*n* = 14)	Placebo (*n* = 14)	Reduced the level of the respiratory quotient, insulin, HOMA, the resting energy expenditure, waist circumference and CRP. Increased % fat oxidation.
Basu et al. [70].	United States	Obesity and metabolic syndrome subjects	25–63 years	Received green tea 4 cups per day containing 440 mg EGCG (*n* = 13), or green tea extract 2 capsules per day containing 460 mg EGCG and 4 cups of water (*n* = 10) for 8 weeks	Receive 4 cups of water per day for 8 weeks (*n* = 12)	Both treatments reduced body weight and body mass index. Green tea beverages showed a decreasing trend in LDL-cholesterol and LDL/HDL-cholesterol ratio versus controls. Green tea beverage also significantly decreased malondialdehyde (MDA) and hydroxynonenal (HNE) levels
Alves Ferreir et al. [71]	Brazil	Overweight without diabetes women.	20–45 years	Receive 1000 mg green tea extract containing 560 mg polyphenols (*n* = 32), receive 1000 mg metformin (*n* = 28), receive 1000 mg green tea extract containing 560 mg polyphenols and 1000 mg metformin (*n* = 31) for 12 weeks.	Receive 1000 mg cellulose (*n* = 29)	The group received 1000 mg green tea extract were reduced the level of fasting glucose, total cholesterol and LDL-c.
Quinhoneiro et al. [72].	Brazil	Women with obesity grade III.	34.5 years	Receive 450 mg EGCG per day for 8 weeks (*n* = 11).	No intervention (*n* = 10).	Upregulated the *UCP3* gene but did not promote weight loss. Did not alter the expression of *UCP1*, *PLIN1*, *PPARG2* and *ADRB3*.
Chen et al. [74].	Taiwan, China	Central obesity women.	44 years	Received a tablet of EGCG at 856.8 mg daily for 12 weeks. (*n* = 39)	Placebo (*n* = 38)	Promoted weight loss, reduced waist circumference, and a consistent decrease in total cholesterol and LDL-cholesterol.
Brown et al. [75].	United Kingdom	Overweight or obese male subjects	40–65 years	Receive 800 mg EGCG per day for 8 weeks. (*n* = 46)	Placebo (*n* = 42)	Did not affect insulin sensitivity, insulin secretion or glucose tolerance but did reduce diastolic blood pressure
Dostal et al. [76].	United States	Overweight and obese postmenopausal women.	61 years	Receive green tea extract containing EGCG at 843 mg daily for 12 months. (*n* = 117)	Placebo (*n* = 120)	Did not change energy intake, body weight, BMI, or waist circumference. No differences were seen in circulating leptin, ghrelin, adiponectin, or glucose concentrations at month 12. Did not affect post-prandial responses of leptin, ghrelin, adiponectin, and satiety.
Dostal et al. [77].	United States	Overweight and obese postmenopausalwomen.	61 years	Receive green tea extract containing EGCG at 843 mg daily for 11–12 months (*n* = 30).	Placebo (*n* = 30).	Did not affect post-prandial responses of leptin, ghrelin, adiponectin, and satiety, though it may be involved in the post-meal insulinemic response.
Dostal et al. [78]	United States	Overweight and obese postmenopausal women.	50–70 years	Receive green tea extract containing EGCG at 843 mg daily for 12 months. (*n* = 61)	Placebo (*n* = 60)	Did not affect adiposity or improve bone mineral density. No changes were seen in circulating leptin, ghrelin, adiponectin, or insulin concentrations. *COMT* genotype did not modify the effect of GTE on any variable.
Huang et al. [79].	Taiwan, China	Obese and high level of LDL-C women.	53.1 years	Receive 856.8 mg EGCG per day for 6 weeks, after 14 days of washout period, switched to placebo for 6 weeks (*n* = 36).	Receive placebo for 6 weeks, after 14 days of washout period, switched to 856.8 mg EGCG per day for 6 weeks (*n* = 37).	Reduced the level of serum LDL-C and increased the level of leptin.
Nicoletti et al. [80].	Brazil	Women with severe obesity.	35.1 years	Received 901.4 mg EGCG per day for 8 weeks (*n* = 11).	-	Upregulated the *RICTOR* and *HIF1-α* expression, however, did not modify *PI3K* expression. Did not change body weight, BMI and fat mass.
Most et al. [82].	The Netherlands	Overweight and obese subjects.	38 ± 2 years	Received a tablet of EGCG at 282 mg and resveratrol at 80 mg day for 12 weeks (*n* = 18)	Placebo (*n* = 20)	Did not significantly affect plasma metabolic profile, whole-body fat mass or energy expenditure.
Most et al. [83].	The Netherlands	Overweight and obese subjects.	36 years	Received a tablet of EGCG at 282 mg and resveratrol at 80 mg day for 12 weeks. (*n* = 11)	Placebo (*n* = 14)	Reduced the expression of pathways related to energy metabolism, oxidative stress, inflammation, and immune defense. Also, blocking cell circle and apoptosis.
Most et al. [84].	The Netherlands	Overweight and obese subjects.	37.8 ± 1.6 years	Received a tablet of EGCG at 282 mg and resveratrol at 80 mg day for 12 weeks. (*n* = 18)	Placebo (*n* = 19)	Reduced bacteroidetes and tended to decrease Faecalibacterium prausnitzii in men but not in women. Moreover, baseline Bacteroidetes abundance was predictive for the combination treatment-induced increase in fat oxidation in men but not in women.
Bagheri et al. [85].	Iran	Overweight women.	38.36 years	Received 500 mg green tea extract containing 45% EGCG per day and endurance training for 8 weeks (*n* = 10).	Endurance training + placebo (*n* = 10); no exercise + placebo (*n* = 10).	The reduction of values including body weight, body index, waist to hip ratio, adiponectin, hs-CRP and body fat percentage in the endurance training and green tea group was significantly higher than endurance training and placebo group interventions. Increased the level of adiponectin.
Bagheri et al. [86]	Iran	Overweight middle-aged males.	40–50 years	Received 500 mg green tea extract containing 45% EGCG per day and endurance training for 8 weeks (*n* = 5).	Endurance training + placebo (*n* = 5); no exercise + placebo (*n* = 5).	The reduction of values including body weight, body index, body fat percentage (BFP), visceral fat area, IL-6 and hs-CRP in the endurance training and green tea group was significantly higher than endurance training and placebo group interventions. Increased the level of adiponectin.
Hill et al. [87]	Australia	Overweight/obese post-menopausal women	45–70 years	Receive 300 mg EGCG per day and regular aerobic exercise for 12 weeks (*n* = 19)	Receive placebo and regular aerobic exercise (*n* = 19)	Reduced heart rate and plasma glucose.

### 3.2. The Impact of Supplemental EGCG Intake on NAFLD

To date, there have been relatively few studies focused on the effects of EGCG in NAFLD, with searches of the PubMed and Web of Science databases returning just four relevant clinical studies (Table 2). Akata et al. performed a 12-week randomized controlled trial wherein 17 individuals with NAFLD were randomly assigned to consume green tea beverages with different catechin doses. Relative to individuals in the placebo control group (0 mg catechins/700 mL) and those receiving low catechin doses (200 mg/700 mL), patients in the high-dose group (1080 mg/700 mL) presented with significant reductions in body fat levels, serum ALT levels, urinary 8-prostaglandin excretion, and liver–spleen computed tomography (CT) attenuation ratios [88]. High-dose catechin intake may thus reduce hepatic adiposity, inflammation, and oxidative stress in individuals with NAFLD. Pezeshki et al. examined the impact of green tea extract consumption on the serum ALT, AST, and ALP levels in 80 NAFLD patients assigned at random to the green tea extract (500 mg/day, 31.429% EGCG) or placebo control. Following a 12-week intervention period, markedly lower serum AST and ALT concentrations were observed in participants receiving green tea extract relative to baseline, while both groups exhibited reductions in serum ALP levels. No comparisons in these values between the groups were performed on completion of the study, however [89]. Hussian et al. similarly treated 80 NAFLD patients with either 500 mg green tea extract capsules (31.429% EGCG) or placebo control twice per day for 12 weeks. Relative to placebo treatment, green tea extract intake was associated with significantly reduced body weight and BMI values, lower serum ALT, AST, hs-CRP, and lipid levels, and enhanced insulin resistance. Ultrasonography also revealed a 67.5% reduction in fatty liver indications after consumption of green tea extract, relative to just 25% in the placebo group [90]. However, a meta-analysis showed that the effect of EGCG on liver enzymes depends on the individual’s health status. In patients with NAFLD, EGCG tends to moderately reduce liver enzyme levels, while in healthy individuals, it may lead to a slight increase [91]. Future studies should take this into account when selecting participants. The most recent report that focused on how EGCG affects NAFLD patients was a randomized controlled trial performed by Yang et al. in which 15 NAFLD patients were enrolled, and outcomes were compared between baseline and the end of a 24-week daily EGCG (300 mg/day) supplementation period. Relative to baseline, these treated patients exhibited significant reductions in hepatic fat content, waist circumference, waist-to-hip ratio, and serum cholesterol together with increased serum HDL-C [40]. The study also indicates that EGCG may improve the progression of NAFLD by reducing serum dipeptidyl peptidase 4 (DPP4) levels. Elevated levels of DPP4 in the liver promote the occurrence and progression of NAFLD. Liver-specific DPP4 knock-in mice exhibit increased weight, higher adiposity, hepatic steatosis, liver injury, adipose inflammation, and elevated cholesterol levels [92].

## 4. The Safety of EGCG

While there are many potentially beneficial effects associated with dietary EGCG supplementation, at high doses it may be associated with certain health risks. Notably, there have been several case reports detailing instances of liver damage linked to green tea extract intake (Table 3). For instance, Patel et al. reported a 16-year-old male from Spain who had been taking the Applied Nutrition^®^ Green Tea Fat Burner supplement containing an EGCG dose of 400 mg/day in an effort to lose weight for 60 days before being hospitalized. While the patient had lost 56 pounds over this period, he was ultimately admitted to the hospital for acute liver injury and potential liver failure. On admission, laboratory testing revealed abnormal findings, while liver histology exhibited diffuse portal and lobular mixed inflammatory cell infiltration, together with evidence of acute and chronic inflammatory activity, hepatocellular necrosis, and cholestasis. As these histological findings were consistent with prior reports of green tea extract-related toxicity, following the exclusion of other potential causes, the hepatic damage in this case was proposed to be attributable to the green tea fat burner supplement [93]. Surapaneni et al. described another case in which a patient suffered from green tea extract-induced acute liver injury. On admission, this patient presented with abnormal liver function test results, and severe scleral icterus was evident on examination of the abdomen. The patient’s BMI was 24.4, their abdomen was soft and not tender, and they did not exhibit any evidence of obesity. No significant abnormalities were detected via abdominal ultrasonography, but magnetic resonance cholangiopancreatography (MRCP) exhibited edema around the distal common bile duct, without apparent stenosis. Two days following admission, the patient developed more severe nausea and vomiting. Further investigation revealed that the patient consumed a green tea extract-containing over-the-counter nutritional supplement (Vital Stem) daily for 1 month before hospitalization. After other possible causes of acute liver injury had been excluded, the green tea extract was considered the most likely cause of the severe hepatic necrosis observed in this case [94]. Gavrić et al. described two cases of acute liver injury among patients consuming dietary supplements containing green tea extract for the purposes of weight loss [95]. Grajecki et al. reported a case of a patient who received a high dose of a GET mixture 72 and 48 h prior to admission and developed jaundice 24 h prior to admission. A physical examination revealed slight liver enlargement with abdominal pain. After excluding other causes, the GET mixture was considered to be the main cause of liver injury in patients [96]. Weinstein et al. described the case of a patient harboring the *α-1 antitrypsin* MZ phenotype who suffered from liver injury, focal necrosis, and parenchymal loss after consuming 4 Slim Quick™ capsules (containing 540 mg EGCG overall) per day [97].

After reviewing the data of 38 interventional studies of EGCG, the European Food Safety Authority’s Panel on Food Additives and Nutrient Sources Added to Food determined that exposure to high doses of EGCG (≥800 mg/day) for extended periods (≥4 weeks) may lead to elevated serum ALT and AST levels in some individuals [98]. A 1-year study of 500 subjects found that 5.1% of these participants experienced moderate-to-severe hepatic dysfunction following daily EGCG intake (843 mg/day), for an odds ratio of 7.0 (95% CI: 2.4–20.3, *p* = 0.0002) [99,100]. Acosta et al. determined that the *uridine 5′-diphospho-glucuronosyltransferase 1A4* (*UGT1A4*) genotype may be significantly related to the risk of increases in serum transaminase levels that are clinically relevant in postmenopausal women following high-dose EGCG supplementation (843 mg/day) [101].

## 5. Summary and Future Perspectives

In conclusion, there is a need for additional studies aimed at clarifying the therapeutic value of EGCG as a treatment for obesity and NAFLD. Based on the available data, the supplemental intake of 150–300 mg/day of EGCG for roughly 12 weeks may help normalize blood lipid and glucose levels in individuals who are obese. The influence of such supplementation on other metabolic parameters such as blood pressure, insulin resistance, and body measurements, however, has not been consistent across studies. Indeed, ongoing debate persists regarding the value of low-dose EGCG (150–300 mg/day) as a means of improving the metabolic status of obese individuals alone or in combination with exercise or other polyphenols. Additional long-term clinical studies will be vital to clarify how EGCG impacts obesity-associated metabolic parameters, particularly given the potential for variations across genotypes and as a function of gender.

In NAFLD patients, there is only relatively weak evidence suggesting the potential beneficial effects of EGCG on hepatic fat and aminotransferase levels, with these results primarily coming from inconsistent, small-scale studies that lack corresponding large-scale validation. High-quality studies will be crucial to definitively establish the impact of EGCG on hepatic fat levels and metabolic outcomes in patients with NAFLD, while also seeking to improve the bioavailability of this compound such that the administered dose levels can be reduced.

Despite the promise of EGCG as a mediator of beneficial health outcomes, some concerns also persist that its use at high doses may lead to hepatotoxicity, particularly at doses exceeding 400 mg/day. As EGCG sensitivity may also be influenced by individual genetics, additional research should be conducted to clarify EGCG tolerability and safety in patients with different genetic profiles.

In summary, while the evidence regarding the health benefits of EGCG as a treatment for obesity or NAFLD remains inconclusive, it continues to hold promise as a tool to combat these disorders. Additional long-term, large-scale studies will ultimately be required to fully clarify the biological activity and therapeutic utility of EGCG in these and other clinical settings.

## Figures and Tables

**Table 2:** Clinical studies of epigallocatechin gallate (EGCG) and its impact on NAFLD.

Author	Country	Patients	Average Age	Dose, Intervention Period	Control	Outcomes
Yang et al. [40].	China	NAFLD	38.53 years	Receive 300 mg EGCG per day for 24 weeks. (*n* = 15)	-	Reduced the liver fat content, waist measurement, waist-to-hip ratio, the level of TC, and increased the level of HDL-C.
Sakata et al. [88].	Japen	NAFLD	20–70 years	Received 700 mL green tea per day for 12 weeks respectively containing 1080 mg/700 mL (*n* = 7) or 200 mg/700 mL (*n* = 5) catechins.	Placebo (*n* = 5)	Reduced body fat content, liver–spleen CT attenuation ratio, serum ALT level and urinary 8-prostaglandin excretion.
Pezeshki et al. [89].	Iran	NAFLD	20–50 years	Receive green tea extract 500 mg containing 31.429% EGCG per day for 12 weeks. (*n* = 40)	Placebo (*n* = 40)	Reduced the level of serum AST and ALT.
Hussian et al. [90].	Pakistan	NAFLD	25 years	Receive 2 capsules green tea extract 500 mg containing 31.429% EGCG per day for 12 weeks. (*n* = 40)	Placebo (*n* = 40)	Reduced weight, BMI, HOMA-IR, the level of serum ALT, AST, hs-CRP, LDL-C, TC and TG. Increased the level of HDL-C and adiponectin. Caused a 67.5% regression of fatty liver changes on ultrasound as compared to placebo which is 25% only.

**Table 3:** Cases of liver toxicity caused by EGCG.

Author	Country	Age (Years)	AST	ALT	ALP	GGT	Total Serum Bilirubin (mg/dL)	Direct Serum Bilirubin (mg/dL)	Unconjugated Bilirubin (mg/dL)
Patel et al. [93].	United States	16	2106 (U/L)	2984 (U/L)	186 (U/L)	78 (U/L)	-	-	1.9
Surapaneni et al. [94].	United States	50	1657 (U/L)	1170 (U/L)	113 (U/L)	-	38	32	-
Gavrić et al. [95].	The Republic of Slovenia	51	14,588 (U/L)	12,882 (U/L)	162 (U/L)	110 (U/L)	-	-	-
Gavrić et al. [95].	The Republic of Slovenia	57	1652 (U/L)	3385 (U/L)	242 (U/L)	262 (U/L)	9.1	-	-
Grajecki et al. [96].	Germany	24	2647 (U/L)	3863 (U/L)	57 (U/L)	21 (U/L)	2.1	-	0.7
Weinstein et al. [97].	United States	24	2320 (IU/L)	2615 (IU/L)	200 (IU/L)	-	4.0	-	-
